# Aging-Related Comorbidity Burden Among Women and Men With or At-Risk for HIV in the US, 2008-2019

**DOI:** 10.1001/jamanetworkopen.2023.27584

**Published:** 2023-08-07

**Authors:** Lauren F. Collins, Frank J. Palella, C. Christina Mehta, JaNae Holloway, Valentina Stosor, Jordan E. Lake, Todd T. Brown, Elizabeth F. Topper, Susanna Naggie, Kathryn Anastos, Tonya N. Taylor, Seble Kassaye, Audrey L. French, Adaora A. Adimora, Margaret A. Fischl, Mirjam-Colette Kempf, Susan L. Koletar, Phyllis C. Tien, Ighovwerha Ofotokun, Anandi N. Sheth

**Affiliations:** 1 Division of Infectious Diseases, Emory University School of Medicine, Atlanta, Georgia; 2 Grady Healthcare System, Ponce de Leon Center, Atlanta, Georgia; 3 Division of Infectious Diseases, Northwestern University, Feinberg School of Medicine, Chicago, Illinois; 4 Department of Biostatistics and Bioinformatics, Rollins School of Public Health, Emory University, Atlanta, Georgia; 5 Department of Medicine, University of Texas Health Sciences Center, Houston; 6 Division of Endocrinology, Diabetes, & Metabolism, Johns Hopkins University School of Medicine, Baltimore, Maryland; 7 Department of Epidemiology, Johns Hopkins Bloomberg School of Public Health, Baltimore, Maryland; 8 Duke Clinical Research Institute and Duke University School of Medicine, Durham, North Carolina; 9 Department of Medicine, Albert Einstein College of Medicine, Bronx, New York; 10 SUNY Downstate Health Sciences University, Brooklyn, New York; 11 Georgetown University Medical Center, Washington, DC; 12 Division of Infectious Diseases, CORE Center, Stroger Hospital of Cook County, Chicago, Illinois; 13 School of Medicine and UNC Gillings School of Global Public Health, University of North Carolina at Chapel Hill; 14 Division of Infectious Diseases, University of Miami Miller School of Medicine, Miami, Florida; 15 Schools of Nursing, Public Health and Medicine, University of Alabama at Birmingham; 16 Division of Infectious Diseases, The Ohio State University Medical Center, Columbus; 17 Division of Infectious Diseases, Department of Medicine, University of California, San Francisco; 18 Medical Service, Department of Veterans Affairs, San Francisco, California

## Abstract

**Importance:**

Despite aging-related comorbidities representing a growing threat to quality-of-life and mortality among persons with HIV (PWH), clinical guidance for comorbidity screening and prevention is lacking. Understanding comorbidity distribution and severity by sex and gender is essential to informing guidelines for promoting healthy aging in adults with HIV.

**Objective:**

To assess the association of human immunodeficiency virus on the burden of aging-related comorbidities among US adults in the modern treatment era.

**Design, Setting, and Participants:**

This cross-sectional analysis included data from US multisite observational cohort studies of women (Women’s Interagency HIV Study) and men (Multicenter AIDS Cohort Study) with HIV and sociodemographically comparable HIV-seronegative individuals. Participants were prospectively followed from 2008 for men and 2009 for women (when more than 80% of participants with HIV reported antiretroviral therapy use) through last observation up until March 2019, at which point outcomes were assessed. Data were analyzed from July 2020 to April 2021.

**Exposures:**

HIV, age, sex.

**Main Outcomes and Measures:**

Comorbidity burden (the number of total comorbidities out of 10 assessed) per participant; secondary outcomes included individual comorbidity prevalence. Linear regression assessed the association of HIV status, age, and sex with comorbidity burden.

**Results:**

A total of 5929 individuals were included (median [IQR] age, 54 [46-61] years; 3238 women [55%]; 2787 Black [47%], 1153 Hispanic or other [19%], 1989 White [34%]). Overall, unadjusted mean comorbidity burden was higher among women vs men (3.4 [2.1] vs 3.2 [1.8]; *P* = .02). Comorbidity prevalence differed by sex for hypertension (2188 of 3238 women [68%] vs 2026 of 2691 men [75%]), psychiatric illness (1771 women [55%] vs 1565 men [58%]), dyslipidemia (1312 women [41%] vs 1728 men [64%]), liver (1093 women [34%] vs 1032 men [38%]), bone disease (1364 women [42%] vs 512 men [19%]), lung disease (1245 women [38%] vs 259 men [10%]), diabetes (763 women [24%] vs 470 men [17%]), cardiovascular (493 women [15%] vs 407 men [15%]), kidney (444 women [14%] vs 404 men [15%]) disease, and cancer (219 women [7%] vs 321 men [12%]). In an unadjusted model, the estimated mean difference in comorbidity burden among women vs men was significantly greater in every age strata among PWH: age under 40 years, 0.33 (95% CI, 0.03-0.63); ages 40 to 49 years, 0.37 (95% CI, 0.12-0.61); ages 50 to 59 years, 0.38 (95% CI, 0.20-0.56); ages 60 to 69 years, 0.66 (95% CI, 0.42-0.90); ages 70 years and older, 0.62 (95% CI, 0.07-1.17). However, the difference between sexes varied by age strata among persons without HIV: age under 40 years, 0.52 (95% CI, 0.13 to 0.92); ages 40 to 49 years, −0.07 (95% CI, −0.45 to 0.31); ages 50 to 59 years, 0.88 (95% CI, 0.62 to 1.14); ages 60 to 69 years, 1.39 (95% CI, 1.06 to 1.72); ages 70 years and older, 0.33 (95% CI, −0.53 to 1.19) (*P* for interaction = .001). In the covariate-adjusted model, findings were slightly attenuated but retained statistical significance.

**Conclusions and Relevance:**

In this cross-sectional study, the overall burden of aging-related comorbidities was higher in women vs men, particularly among PWH, and the distribution of comorbidity prevalence differed by sex. Comorbidity screening and prevention strategies tailored by HIV serostatus and sex or gender may be needed.

## Key Points

**Question:**

Is HIV associated with aging-related comorbidity burden differentially among US women and men?

**Findings:**

In this cross-sectional study including 5926 US adults, the overall burden of 10 non-AIDS comorbidities after covariate adjustment was significantly higher in women vs men, particularly among persons with HIV, and comorbidity prevalence differed by sex.

**Meaning:**

Clinical guidance and tools for promoting healthy aging in HIV are urgently needed, and these results suggest that novel strategies developed for comorbidity screening and prevention in persons with HIV would ideally consider sex and gender differences in comorbidity risk and address social determinants of health.

## Teaser

This cross-sectional study of US adults compares the burden of non-AIDS comorbidities between persons with HIV and those without by age and sex.

## Introduction

Antiretroviral therapy (ART) has dramatically improved the lifespan of persons with HIV (PWH), such that more than 50% of US PWH were aged 50 years or older in 2019.^1^ For individuals with access to care, despite the gap in overall life expectancy by HIV status narrowing over 2 decades, the gap in comorbidity-free survival persists: between 2014 and 2016, PWH lived 16.3 fewer healthy years than peers without HIV.^2^ Aging-related non-AIDS comorbidities (NACM) represent a growing health challenge for PWH, in whom comorbidity burden is higher and onset a decade earlier than the general population.^3,4,5,6^ The downstream impact is immense, including reduced quality of life, premature mortality, and increased health care utilization and cost.^7,8^

Emerging data suggest that sex differences exist in NACM risk and severity.^9^ For example, women vs men with HIV have a nearly 2-fold higher risk of cardiovascular events, an observation associated with disproportionate risk among young women with HIV.^10^ In the HIV Outpatient Study, women with HIV had a higher overall number of NACM than men with HIV; however, this finding was attenuated in fully adjusted models.^11^ Sex and gender differences in biologic and sociobehavioral factors uniquely contribute to the experience and outcomes of aging with HIV; and therefore, substantial representation of women in HIV research and sex-stratified analyses should be highly prioritized.^12,13,14^

We previously found that women with HIV had a significantly higher prevalent and incident burden of aging-related comorbidities than women without HIV.^15,16^ Building on these observations, our study combined data from the largest and longest-running observational US cohorts of women and men with and without HIV to assess the association of HIV and comorbidity burden by sex.

## Methods

We analyzed data from the Multicenter AIDS Cohort Study (MACS) and the Women’s Interagency HIV Study (WIHS) prior to the cohorts’ merging in April 2019 to form the MACS/WIHS Combined Cohort Study. Designed to evaluate the natural history of HIV in gay and bisexual men (MACS) and cisgender women (WIHS), these multicenter US prospective observational cohort studies were established in 1983 and 1993, respectively. Each study used a unified protocol to enroll PWH and sociodemographically comparable HIV-seronegative individuals in multiple enrollment waves as previously described.^17^

Participants engaged in semiannual study visits comprising detailed collection of sociodemographics (including self-reported race, ethnicity, and sex), medical history and behaviors, self-reported medication (including ART) use, physical examination (including blood pressure measurement^15^), and biospecimens (including plasma to evaluate kidney and liver function, viral hepatitis, CD4 count, and HIV-1 viral load). Race and ethnicity data are collected in MACS and WIHS given shifts in demographics of the US HIV epidemic over time and disparities in HIV care continuum outcomes by race and ethnicity specifically. The study protocols were approved by each site’s institutional review board; all participants provided written informed consent. This study followed the Strengthening the Reporting of Observational Studies in Epidemiology (STROBE) reporting guideline for cohort studies.

### Study Design

We performed a cross-sectional study focused on the modern ART era, and thus included PWH and HIV-seronegative participants with 2 or more WIHS or MACS study visits since 2009 or 2008, respectively (the calendar year in which more than 80% of PWH reported ART use). Longitudinal data from study enrollment through observation end (visit prior to lost-to-follow-up or March 2019) were cross-sectionalized, such that age, covariates, and NACM prevalence and burden were assessed as of latest study visit for each participant. Participants without data available for all outcomes assessed were excluded.

### Outcome Measures

We evaluated 10 aging-related NACM given their prevalence in the general population and among PWH, association with morbidity and mortality, and ascertainment in the study cohorts. The primary outcome was NACM burden, defined as the number of total NACM per participant out of 10: hypertension, dyslipidemia, diabetes, cardiovascular disease, kidney disease, liver disease, lung disease, bone disease, psychiatric illness, and non-AIDS cancer. Secondary outcomes were prevalence of each NACM, defined as the presence of the condition as of participant’s last observation. Up to 3 data sources were used to define each NACM as previously described^15,16^: self-reported diagnosis or medication, clinical measurement, and/or laboratory evidence. Definitions of NACM were harmonized across the WIHS and MACS so that the same comorbidity criteria and frequency of assessments were used across participants (eTable 1 in Supplement 1). NACM were assigned if a participant met criteria of the comorbidity by latest visit, with the exception of certain NACM defined by parameters with the potential to fluctuate on repeated measure, for which criteria needed to be satisfied on at least 2 consecutive visits (eg, requiring estimated glomerular filtration rate below 60 mL/min/1.73 m^2^ on 2 consecutive visits for kidney disease).

### Statistical Analysis

We compared participant characteristics by sex using χ^2^ tests (categorical variables) and Wilcoxon rank-sum tests (continuous variables). Sex instead of gender was used as an independent variable in analyses given the recruitment criteria of the cohorts. We assessed the association of independent variables (HIV serostatus, age group, sex) with the prevalence of each comorbidity by χ^2^ tests and with NACM burden by 2 sample *t* tests or linear regression. For the entire cohort and then stratified by HIV serostatus, we assessed for a linear trend by ranked age category (ages under 40, 40 to 49, 50 to 59, 60 to 69, and 70 years or older) using unadjusted linear regression for NACM burden.

For the primary outcome (NACM burden), we performed separate linear regression models including the entire cohort (models 1 and 2) and including PWH only (models 3 and 4). Base models assessed the association of independent variables and respective interaction terms on NACM burden: HIV serostatus, age, sex, 2-way interaction terms, and HIV × age × sex (model 1); and age, sex, age × sex (model 3). Building upon models 1 and 3, respectively, covariate-adjusted models included traditional comorbidity risk factors of race and ethnicity, body mass index (BMI), socioeconomic status (SES), use of cigarettes, alcohol, and/or crack or cocaine (models 2 and 4) and HIV-specific factors including CD4 count, CD4 nadir, time since ART initiation, proportion of visits with HIV-1 RNA below 200 copies/mL during observation, recent protease inhibitor use, and recent abacavir use (model 4 only). Model-based estimates of mean NACM burden by HIV, age, and sex categories were determined and estimated mean differences with 95% CI were generated; model fit was assessed through residual plots.

We derived a summary SES covariate that incorporated harmonized variables across the MACS and WIHS to account for differences in how social determinants of health were assessed (eTable 2 in Supplement 1). Other covariates were inherently compatible across the studies.

Analyses were conducted in 2021 using SAS version 9.4 (SAS Institute). Statistical significance (2-sided) was set at α = .05.

## Results

### Participant Characteristics

A total of 5929 participants were included (median [IQR] age, 54 [46-61] years; 2787 Black [47%], 1153 Hispanic or other [19%], 1989 White [34%]) (eFigure 1 in Supplement 1). Cohorts comprised 3238 women (55%) (2316 PWH, 922 HIV-seronegative) and 2691 men (45%) (1452 PWH, 1239 HIV-seronegative) with median (IQR) observation of 16.1 (5.0-18.2) and 25.5 (15.3-34.1) years, respectively. At last observation, women vs men had a median (IQR) age of 51 (44-57) years vs 58 (50-66) years, 2100 (65%) vs 638 (25%) were Black, 2410 (78%) vs 724 (32%) had income below 150% of the federal poverty level, and 1195 (37%) vs 496 (21%) currently used cigarettes, respectively (Table 1). Among women vs men with HIV, median (IQR) CD4 count was 620 (398-864) vs 636 (464-854) cells/mm^3^ and 1850 (81%) vs 840 (86%) had HIV-1 RNA levels below 200 copies/mL, respectively.

**Table 1.** Demographic and Clinical Characteristics of Women and Men Living With and Without HIV

Characteristic^a^	Participants, No. (%)^b^				
	Total (n = 5929)^c^	Women with HIV (n = 2316)	Women without HIV (n = 922)	Men with HIV (n = 1452)	Men without HIV (n = 1239)
Age, median (IQR), y	54 (46-61)	51 (44-57)	50 (41-56)	56 (47-63)	62 (54-69)
Age group, y					
<40	785 (13)	286 (12)	191 (21)	203 (14)	105 (8)
40-49	1280 (22)	677 (29)	269 (29)	230 (16)	104 (8)
50-59	2073 (35)	947 (41)	318 (34)	502 (35)	306 (25)
60-69	1319 (22)	356 (15)	129 (14)	402 (28)	432 (35)
≥70	472 (8)	50 (92)	15 (2)	115 (8)	292 (24)
Observation time, median (IQR), y	16.6 (7.3-24.0)	16.0 (4.9-18.4)	16.2 (5.0-17.9)	16.4 (10.3-32.8)	31.8 (16.0-34.3)
Last calendar year of observation, median (IQR)	2018 (2016-2019)	2018 (2014-2019)	2018 (2016-2019)	2018 (2017-2019)	2018 (2017-2019)
Last calendar year of observation					
2008-2012	713 (12)	365 (16)	122 (13)	93 (8)	111 (9)
2013-2017	1127 (19)	439 (19)	166 (18)	253 (21)	210 (17)
2018-2019	4089 (69)	1497 (65)	634 (69)	852 (71)	918 (74)
Race and ethnicity					
White, non-Hispanic	1989 (34)	274 (12)	83 (9)	756 (52)	876 (71)
Black, non-Hispanic	2787 (47)	1491 (64)	621 (67)	439 (30)	236 (19)
Hispanic	993 (17)	478 (21)	180 (20)	229 (16)	106 (9)
Other^d^	160 (3)	73 (3)	38 (4)	28 (2)	21 (2)
BMI, median (IQR)	28.1 (24.2-33.3)	29.6 (25.1-35.5)	31.4 (26.3-37.6)	25.8 (23.0-29.2)	26.6 (23.8-30.1)
SBP, median (IQR), mm Hg	126 (114-139)	123 (111-138)	127 (115-142)	127 (117-138)	130 (120-141)
DBP, median (IQR), mm Hg	76 (69-84)	75 (68-83)	77 (69-84)	77 (71-84)	77 (70-84)
Antihypertensive medication use	2416 (41)	977 (42)	354 (38)	572 (40)	513 (42)
Lipid-lowering medication use	1683 (29)	466 (20)	167 (18)	562 (40)	488 (41)
eGFR, mL/min/1.73 m^2^ (CKD-EPI)	88.5 (72.0-104.1)	89.9 (71.6-107.4)	98.4 (83.0-112.2)	83.4 (66.6-98.1)	85.2 (72.7-95.7)
CES-D score^e^	8 (3-18)	9 (3-19)	9 (3-17)	9 (3-19)	7 (2-16)
Education					
≤High school	2661 (45)	1519 (66)	579 (63)	372 (26)	191 (15)
>High school	3261 (55)	795 (34)	191 (15)	1080 (74)	1048 (85)
Income					
<150% FPL	3202 (58)	1738 (79)	685 (78)	524 (39)	255 (23)
≥150% FPL	2335 (42)	474 (21)	193 (22)	803 (61)	865 (77)
Employed	2631 (46)	815 (35)	407 (44)	733 (53)	676 (57)
Insured	5416 (95)	2259 (98)	770 (84)	1295 (96)	1092 (97)
Cigarette use					
Never	1770 (31)	786 (34)	233 (25)	387 (29)	364 (31)
Current	1733 (30)	792 (34)	408 (44)	340 (25)	193 (17)
Former	2251 (39)	737 (32)	280 (30)	624 (46)	610 (52)
Current alcohol use					
None	2350 (42)	1360 (59)	420 (46)	335 (26)	235 (21)
1-7 Drinks/wk	2517 (45)	793 (35)	373 (41)	741 (58)	610 (55)
>7 Drinks/wk	734 (13)	143 (6)	127 (14)	198 (16)	266 (24)
Marijuana use					
Never	1506 (26)	781 (34)	200 (22)	286 (21)	239 (20)
Current	1575 (27)	451 (20)	240 (26)	507 (37)	377 (32)
Former	2693 (47)	1077 (47)	481 (52)	567 (42)	568 (48)
Crack or cocaine use					
Never	2970 (51)	1333 (58)	427 (46)	596 (44)	614 (52)
Current	406 (7)	138 (6)	77 (8)	118 (9)	73 (6)
Former	2402 (42)	840 (36)	417 (45)	648 (48)	497 (42)
Opioid use (heroin or methadone)					
Never	4760 (82)	1884 (82)	686 (74)	1152 (85)	1038 (88)
Current	118 (2)	38 (2)	19 (2)	38 (3)	23 (2)
Former	897 (16)	389 (17)	216 (23)	170 (13)	122 (10)
Injection drug use					
Never	4856 (84)	1876 (81)	757 (82)	1137 (84)	1086 (92)
Current	69 (1)	19 (1)	8 (1)	33 (2)	9 (1)
Former	848 (15)	416 (18)	156 (17)	189 (14)	87 (7)
Chronic HBV	233 (4)	56 (2)	10 (1)	118 (8)	49 (4)
Chronic HCV	624 (11)	306 (13)	87 (9)	168 (12)	63 (5)
CD4 cell count, median (IQR), cells/mm^3^	NA	620 (398-864)	NA	636 (464-854)	NA
CD4 cell count, cells/mm^3^					
≥500	NA	1456 (64)	NA	897 (70)	NA
<500	NA	829 (36)	NA	376 (30)	NA
CD4 nadir, median (IQR), cells/mm^3^	NA	281 (160-415)	NA	311 (194-446)	NA
CD4 nadir, cells/mm^3^					
≥200	NA	1511 (68)	NA	1069 (74)	NA
<200	NA	718 (32)	NA	373 (26)	NA
HIV viral load, copies/mL					
Suppressed^f^	NA	1850 (81)	NA	840 (86)	NA
200-999	NA	101 (4)	NA	33 (3)	NA
≥1000	NA	327 (14)	NA	101 (10)	NA
Proportion visits HIV suppressed^f^					
From baseline visit	NA	69.3 (44.6-93.3)	NA	72.7 (50-90.5)	NA
2008-2009 or later	NA	90 (61.1-100)	NA	100 (76.2-100)	NA
Proportion visits HIV suppressed^f^					
≥90%	NA	665 (29)	NA	379 (26)	NA
<90%	NA	1651 (71)	NA	1071 (74)	NA
Time since ART initiation, median (IQR), y	NA	12.9 (7.8-17.6)	NA	15.4 (7.7-20.5)	NA
Time since ART initiation, y					
<5	NA	263 (11)	NA	164 (11)	NA
5 to <10	NA	559 (24)	NA	283 (19)	NA
10 to <15	NA	539 (23)	NA	226 (16)	NA
≥15	NA	887 (38)	NA	732 (50)	NA
Never initiated ART	NA	68 (3)	NA	47 (3)	NA
Antiretroviral class^g^					
PI	NA	704 (30)	NA	372 (26)	NA
NNRTI	NA	532 (23)	NA	391 (27)	NA
INSTI	NA	718 (31)	NA	498 (34)	NA
Other	NA	186 (8)	NA	94 (6)	NA
Not receiving therapy	NA	176 (8)	NA	97 (7)	NA
Specified NRTI^h^					
Abacavir	NA	450 (19)	NA	256 (18)	NA
TDF	NA	1067 (46)	NA	418 (30)	NA
TAF	NA	598 (26)	NA	570 (40)	NA
Antiretroviral adherence					
≥95%	NA	1741 (82)	NA	1164 (90)	NA
<95%	NA	389 (18)	NA	133 (10)	NA

Abbreviations: ART, antiretroviral therapy; BMI, body mass index (calculated as weight in kilograms divided by height in meters squared); CES-D, Center for Epidemiologic Studies Depression; CKD-EPI, Chronic Kidney Disease Epidemiology Collaboration; DBP, diastolic blood pressure; eGFR, estimated glomerular filtration rate; FPL, federal poverty level; HBV, hepatitis B virus; HCV, hepatitis C virus; INSTI, integrase strand transfer inhibitor; MACS, Multicenter AIDS Cohort Study; NA, not applicable; NRTI, nucleoside reverse transcriptase inhibitor; NNRTI, non-nucleoside reverse transcriptase inhibitor; PI, protease inhibitor; SBP, systolic blood pressure; TAF, tenofovir alafenamide fumarate; TDF, tenofovir disoproxil fumarate; WIHS, Women’s Interagency HIV Study.

^a^ Percentages of columns may not total 100 due to rounding.

^b^ χ^2^ test performed for categorical variables and Wilcoxon rank sum for continuous variables.

^c^ Data missing for the following: BMI (518 participants); SBP (478 participants); DBP (478 participants); eGFR (407 participants); CES-D (219 participants); CD4 cell count (210 participants); CD4 nadir (97 participants); proportion of visits suppressed (2 participants); proportion of visits suppressed 2008-2009 or later in WIHS/MACS (50 participants); time since ART initiation (117 participants).

^d^ Other included Asian and Pacific Islander, Native American and Alaskan native, and other non-Hispanic groups.

^e^ Range, 0-60; threshold for depressive symptoms, 16 or greater.

^f^ HIV viral load less than 200 copies/mL and/or below the lower limit of quantification of assay.

^g^ Categorized hierarchically as PI superior to NNRTI superior to INSTI superior to other.

^h^ Of those receiving any NRTI, not mutually exclusive.

### NACM Burden and Prevalence

**Figure 1.**
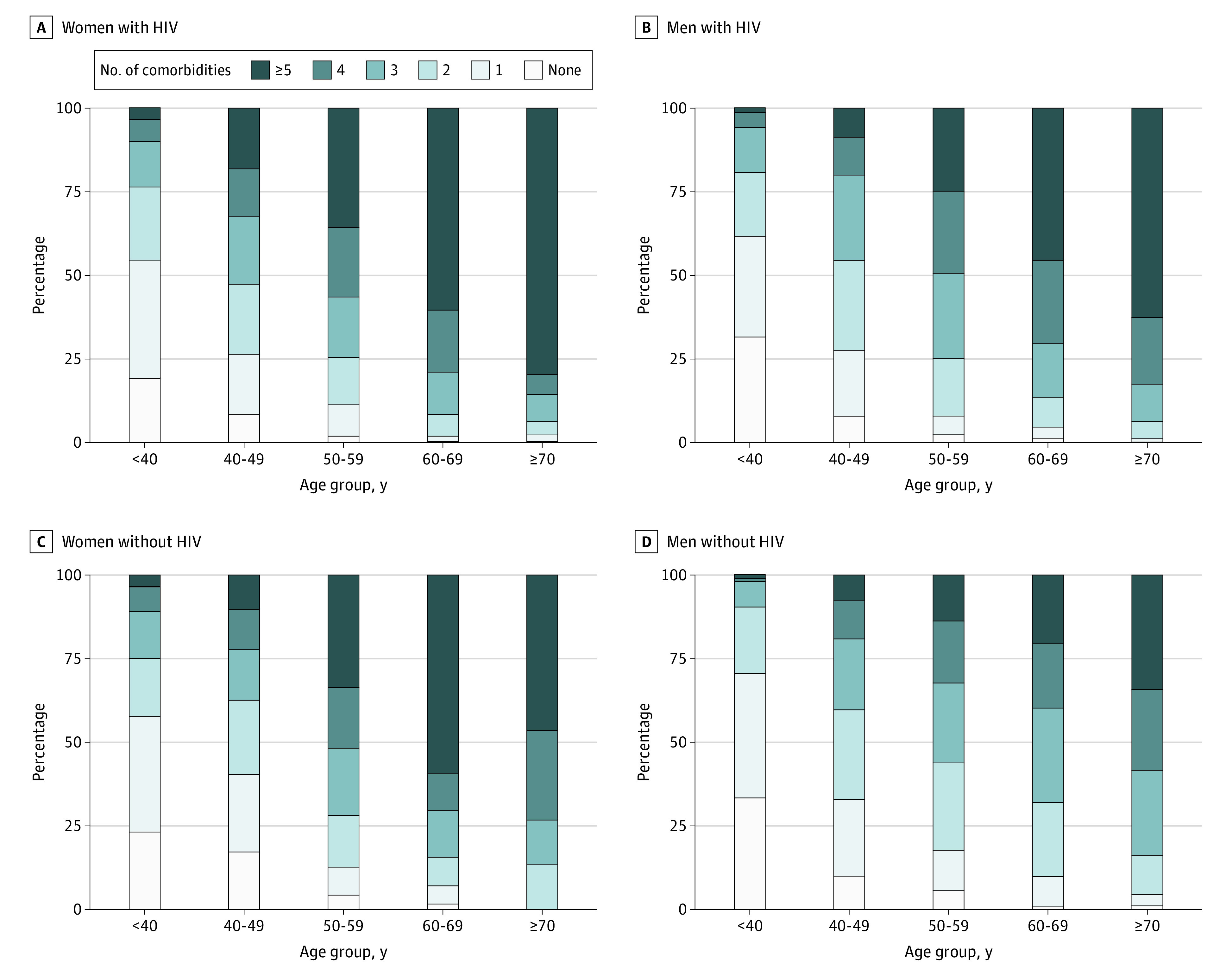
Distribution of Prevalent Non-AIDS Comorbidity (NACM) Burden by HIV Serostatus, Sex, and Age Group The burden of comorbidities was associated with increasing age; with persons living with vs without HIV; and with women, regardless of HIV serostatus.

Figure 1 shows the distribution of NACM burden by HIV serostatus, age group, and sex; eFigure 2 in Supplement 1 additionally stratifies the distribution of NACM burden by race and ethnicity. Overall, increasing age category was associated with progressively higher mean (SD) NACM burden: age under 40 years, 1.49 (1.32); ages 40 to 49 years, 2.58 (1.72); ages 50 to 59 years, 3.63 (1.80); ages 60 to 69 years, 4.21 (1.81); ages 70 years or older, 4.41 (1.73) (*P* < .001). PWH vs persons without HIV had a higher mean (SD) NACM burden overall (3.47 [1.99] vs 3.03 [1.86]; *P* < .001) and in every age category: age under 40 years, 1.52 (1.32) vs 1.42 (1.33); ages 40 to 49 years, 2.73 (1.73) vs 2.21 (1.63); ages 50 to 59 years, 3.77 (1.77) vs 3.31 (1.82); ages 60 to 69 years, 4.65 (1.75) vs 3.60 (1.71); ages 70 years or older, 5.27 (1.73) vs 3.95 (1.54). Overall, women had a higher mean (SD) NACM burden than men (3.36 [2.08] vs 3.24 [1.79]; *P* = .02) although the differences were not statistically significant among PWH (3.51 [2.06] vs 3.40 [1.88]; *P* = .07) or persons without HIV (2.99 [2.09] vs 3.06 [1.66]; *P* = .37).

Overall, specific NACM prevalence ranged from 540 of 5929 participants (9%) diagnosed with cancer to 4214 (71%) with hypertension. Women vs men had a higher prevalence of bone disease (1364 women [42%] vs 512 men [19%]), lung disease (1245 women [38%] vs 259 men [10%]), and diabetes (763 women [24%] vs 470 men [17%]), but a lower prevalence of hypertension (2188 women [68%] vs 2026 men [75%]), psychiatric illness (1771 women [55%] vs 1565 men [58%]), dyslipidemia (1312 women [41%] vs 1728 men [64%]), liver disease (1093 women [34%] vs 1032 men [38%]), and non-AIDS cancer (219 women [7%] vs 321 men [12%]); cardiovascular (493 women [15%] vs 407 men [15%]) and kidney disease (444 women [14%] vs 404 men [15%]) prevalence did not significantly differ by sex (eTable 3 in Supplement 1). eTable 4 in Supplement 1 details the age-stratified prevalence and burden of NACM among women vs men.

### Estimated NACM Burden by HIV Serostatus and Age Group

Among all participants, the estimated mean difference in NACM burden (model 1) was significantly greater for women vs men in every age strata among PWH: age under 40 years, 0.33 (95% CI, 0.03-0.63); ages 40 to 49 years, 0.37 (95% CI, 0.12-0.61); ages 50 to 59 years, 0.38 (95% CI, 0.20-0.56); ages 60 to 69 years, 0.66 (95% CI, 0.42-0.90); ages 70 years and older, 0.62 (95% CI, 0.07-1.17). However, the NACM burden differed for women vs men by age strata among persons without HIV: age under 40 years, 0.52 (95% CI, 0.13 to 0.92); ages 40 to 49 years, −0.07 (95% CI, −0.45 to 0.31); ages 50 to 59 years, 0.88 (95% CI, 0.62 to 1.14); ages 60 to 69 years, 1.39 (95% CI, 1.06 to 1.72); ages 70 years and older, 0.33 (95% CI, −0.53 to 1.19) (HIV × age × sex, *P* for interaction = .001) (Table 2). There was not a significant trend in the estimated mean difference in NACM burden by sex with advancing age among PWH nor among persons without HIV.

**Table 2.** Estimated Mean NACM Burden Among Women and Men by HIV Serostatus, Sex, and Age Group

HIV serostatus	Age, y	Estimated mean NACM burden (95% CI)		*P* value for comparison
		Women	Men	
**Model 1** ^a^				
Positive	<40	1.66 (1.47-1.85)	1.33 (1.10-1.56)	.03
	40-49	2.82 (2.69-2.94)	2.45 (2.24-2.67)	.004
	50-59	3.90 (3.79-4.00)	3.52 (3.37-3.67)	<.001
	60-69	5.00 (4.83-5.18)	4.34 (4.18-4.50)	<.001
	≥70	5.70 (5.24-6.16)	5.08 (4.77-5.38)	.03
Negative	<40	1.61 (1.37-1.84)	1.09 (0.77-1.40)	.01
	40-49	2.19 (1.99-2.39)	2.26 (1.94-2.58)	.72
	50-59	3.75 (3.56-3.93)	2.86 (2.68-3.05)	<.001
	60-69	4.67 (4.39-4.96)	3.28 (3.13-3.44)	<.001
	≥70	4.27 (3.43-5.12)	3.94 (3.75-4.13)	.46
**Model 2** ^b^				
Positive	<40	1.67 (1.47-1.88)	1.56 (1.31-1.81)	.48
	40-49	2.73 (2.59-2.87)	2.55 (2.31-2.79)	.18
	50-59	3.66 (3.53-3.78)	3.61 (3.44-3.78)	.61
	60-69	4.75 (4.56-4.93)	4.44 (4.24-4.63)	.02
	≥70	5.51 (5.02-5.99)	5.25 (4.91-5.59)	.39
Negative	<40	1.60 (1.36-1.84)	1.37 (1.02-1.72)	.29
	40-49	2.17 (1.96-2.37)	2.34 (1.99-2.70)	.39
	50-59	3.45 (3.26-3.63)	3.00 (2.79-3.21)	.002
	60-69	4.27 (3.98-4.56)	3.40 (3.21-3.59)	<.001
	≥70	4.15 (3.32-4.99)	4.07 (3.83-4.31)	.85

Abbreviation: NACM, non-AIDS comorbidity.

^a^ Linear regression model including HIV, age, sex, and all interaction terms in the model (HIV × sex, *P *for interaction = .30; HIV × age, *P* for interaction < .001; age × sex, *P* for interaction < .001; HIV × age × sex, *P* for interaction = .001). The model uses 5929 observations.

^b^ Adjusted linear regression model including HIV, age, sex, and all interaction terms in the model (HIV × sex *P* for interaction = .44; HIV × age *P* for interaction < .001; age × sex, *P* for interaction = .006; HIV × age × sex, *P* for interaction = .04) in addition to race (*P* < .001), body mass index (*P* < .001), socioeconomic status (*P* < .001), cigarette use (*P* < .001), current alcohol use (*P* < .001), and crack or cocaine use (*P* < .001). The model uses 5192 observations with complete data (737 of 5929 were excluded).

**Figure 2.**
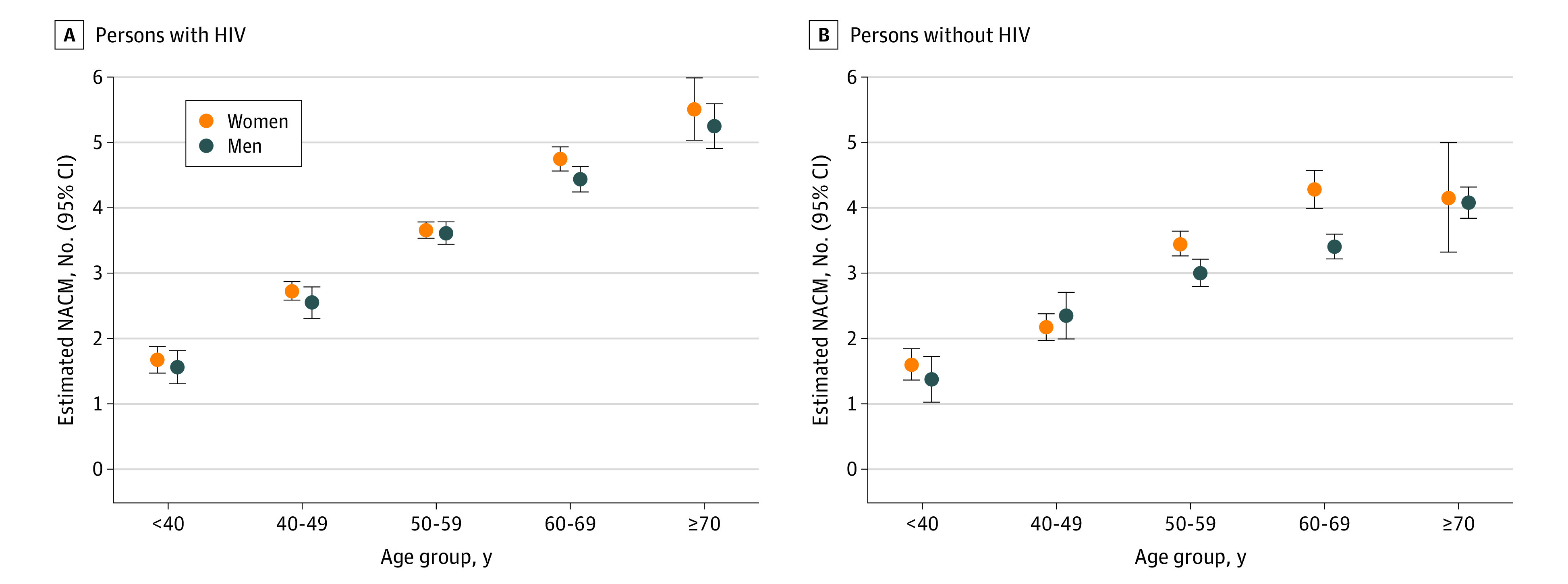
Estimated Mean Number of Non-AIDS Comorbidities (NACM) Among Persons With and Without HIV Stratified by Sex and Age Group Participants were enrolled in the Women’s Interagency HIV Study (for women) or the Multicenter AIDS Cohort Study (men), stratified by sex and age group. Adjusted linear regression (model 2) was performed with the following covariates included: race and ethnicity, body mass index, socioeconomic status, cigarette use, alcohol use, crack or cocaine use, in addition to HIV serostatus, age, sex, and all interaction terms (HIV × age × sex, *P* for interaction = .04).

When adjusting for covariates (model 2), findings were attenuated but HIV and age still significantly modified the estimated mean NACM burden by sex (HIV × age × sex, *P *for interaction = .04) (Table 2 and Figure 2). In this model, the age-stratified estimated mean NACM burden ranged from 1.67 (95% CI, 1.47-1.88) for individuals under age 40 years to 5.51 (95% CI, 5.02-5.99) for those ages 70 years or older among women with HIV; from 1.56 (95% CI, 1.31-1.81) to 5.25 (95% CI, 4.91-5.59) among men with HIV; 1.60 (95% CI, 1.36-1.84) to 4.15 (95% CI, 3.32-4.99) among women without HIV; and from 1.37 (95% CI, 1.02-1.72) to 4.07 (95% CI, 3.83-4.31) among men without HIV. Furthermore, estimated mean NACM burden was significantly associated with race, BMI, SES, current use of cigarettes, alcohol, and cocaine (Table 2).

### Estimated NACM Burden Among PWH

Among PWH specifically, the estimated mean difference in NACM burden was significantly greater for women vs men in every age strata; however, this interaction was not significant (model 3; age × sex, *P* for interaction = .29) (eTable 5 in Supplement 1). When adjusting for traditional and HIV-specific covariates (model 4), the estimated mean difference in NACM burden for women vs men by age group was 0.03 for age under 40 years (95% CI, −0.30 to 0.36), 0.11 for ages 40 to 49 years (95% CI, −0.16 to 0.39), 0.28 for ages 50 to 59 years (95% CI, 0.08 to 0.48), 0.60 for ages 60 to 69 years (95% CI, 0.32 to 0.87), 0.52 for ages 70 years and older (95% CI, −0.08 to 1.11); age significantly modified the association of sex and NACM burden (age × sex, *P* for interaction = .04) (eTable 5 in Supplement 1).

Univariable and multivariable results of the estimated mean NACM burden among PWH stratified by each of the covariates included in model 4 are included in eTable 6 in Supplement 1 and Table 3, respectively. After adjustment, individuals ages 70 years or older vs younger than 40 years had an estimated mean (SE) 2.95 (0.18) more comorbidities, and women with HIV had a 0.31 (0.09) higher comorbidity count vs men with HIV; significant differences were also observed by strata of race, BMI, cigarette and cocaine use, time since ART initiation, proportion of visits with HIV-1 RNA below 200 copies/mL, and recent abacavir use (Table 3).

**Table 3.** Multivariable Analysis of Risk Factors Associated With Prevalent Burden of NACM Among Persons Living With HIV

Risk factor	Estimated mean No. of NACM (95% CI)	β (SE)^a^	*P* value^b^
Sex^c^			
Women	3.63 (3.46-3.79)	0.31 (0.09)	.001
Men	3.32 (3.16-3.48)	[Reference]	
Age group, y^c^			
≥70	4.91 (4.59-5.23)	2.95 (0.18)	<.001
60-69	4.28 (4.10-4.45)	2.32 (0.11)	
50-59	3.50 (3.35-3.64)	1.54 (0.10)	
40-49	2.72 (2.55-2.89)	0.76 (0.11)	
<40	1.96 (1.76-2.16)	[Reference]	
Race			
Black, non-Hispanic	3.29 (3.14-3.44)	−0.50 (0.08)	<.001
Hispanic or other^d^	3.34 (3.16-3.51)	−0.46 (0.09)	
White, non-Hispanic	3.79 (3.63-3.96)	[Reference]	
BMI			
≥30	3.60 (3.44-3.76)	0.26 (0.06)	<.001
<30	3.34 (3.21-3.48)	[Reference]	
Socioeconomic status^e^			
Level 1 (low)	3.94 (3.78-4.10)	0.81 (0.09)	<.001
Level 2	3.11 (2.91-3.30)	−0.02 (0.11)	
Level 3	3.70 (3.53-3.88)	0.58 (0.09)	
Level 4	3.50 (3.30-3.69)	0.37 (0.10)	
Level 5 (high)	3.13 (2.94-3.31)	[Reference]	
Cigarette use			
Current	3.71 (3.55-3.87)	0.51 (0.08)	<.001
Former	3.52 (3.37-3.68)	0.33 (0.07)	
Never	3.19 (3.03-3.36)	[Reference]	
Current alcohol use			
>7 Drinks/wk	3.38 (3.17-3.59)	−0.21 (0.11)	.05
1-7 Drinks/wk	3.46 (3.32-3.60)	−0.13 (0.06)	
None	3.59 (3.43-3.74)	[Reference]	
Crack or cocaine use			
Current	3.47 (3.23-3.71)	0.17 (0.12)	<.001
Former	3.64 (3.50-3.79)	0.33 (0.06)	
Never	3.31 (3.17-3.44)	[Reference]	
CD4 count, cells/mm^3^			
<500	3.49 (3.33-3.65)	0.03 (0.06)	.59
≥500	3.46 (3.32-3.60)	[Reference]	
CD4 nadir, cells/mm^3^			
<200	3.49 (3.33-3.65)	0.03 (0.06)	.65
≥200	3.46 (3.32-3.60)	[Reference]	
Time since ART initiation, y			
≥15	3.96 (3.81-4.11)	0.99 (0.10)	<.001
10 to <15	3.64 (3.47-3.81)	0.67 (0.10)	
5 to <10	3.33 (3.17-3.50)	0.37 (0.10)	
<5 or never initiated ART	2.97 (2.76-3.17)	[Reference]	
Proportion visits HIV suppressed from initial study visit^f^			
<90%	3.65 (3.52-3.78)	0.36 (0.07)	<.001
≥90%	3.30 (3.13-3.46)	[Reference]	
PI use in last 6 mo			
Yes	3.53 (3.37-3.69)	0.12 (0.06)	.07
No	3.42 (3.28-3.55)	[Reference]	
Abacavir use in last 6 mo			
Yes	3.61 (3.44-3.79)	0.28 (0.07)	<.001
No	3.33 (3.20-3.46)	[Reference]	

Abbreviations: ART, antiretroviral therapy; BMI, body mass index (calculated as weight in kilograms divided by height in meters squared); PI, protease inhibitor.

^a^ Estimated mean difference in NACM.

^b^ Model 4: adjusted linear regression performed including all covariates listed in addition to age × sex interaction (*P* = .04). Model uses 3236 observations with complete data (263 of 3499 were excluded).

^c^ Adjusted for age × sex interaction (*P* = .04)**.**

^d^ Other included Asian and Pacific Islander, Native American and Alaskan native, and other non-Hispanic groups.

^e^ Socioeconomic status determined by participant’s reported educational level, federal poverty level, and employment status; graded from level 1 (low) to level 5 (high).

^f^ HIV viral load <200 copies/mL and/or below the lower limit of quantification of assay.

## Discussion

Leveraging data from the largest and longest US observational cohort study of persons with and without HIV, we found that among nearly 6000 participants with a median observation of 17 years, the burden of 10 aging-related NACM was higher in women vs men overall and particularly among PWH. The most prevalent NACM were hypertension, psychiatric illness, dyslipidemia, liver disease, and bone disease; and the distribution of prevalent NACM differed by sex. HIV serostatus and age significantly modified the estimated NACM burden by sex, but results were attenuated when adjusting for traditional comorbidity risk factors. Our findings suggest that HIV is associated with sex or gender differences in aging-related comorbidity development, which has important implications for developing and/or refining NACM screening and prevention strategies to promote healthy aging in this population.

Multimorbidity, the presence of 2 or more chronic conditions, represents a growing public health threat.^18,19^ Multimorbidity prevalence is rising in the US, leading to concerns that its impact on health-related quality of life, health care utilization and costs, and mortality, which is already substantial, may be amplified.^20,21,22^ In population studies across varied high-income settings, female sex has emerged as a risk factor for multimorbidity development and progression.^21,23,24,25^ In these studies, multimorbidity was associated with obesity, mental health disorders, substance use, and lower SES,^23,24,25^ variables often differing by sex and gender^26^ and exacerbated in women with HIV.^27^ In this study of the WIHS/MACS cohorts, we found that NACM burden was significantly higher in women vs men overall (3.4 vs 3.2) and among PWH specifically, but not among HIV-seronegative peers. Our analysis adds to existing research on sex differences in multimorbidity prevalence by specifically examining the potential role of HIV and by substantially representing women, ie, over 50% of the study population vs less than 25% in prior studies involving PWH.^11,28^ While the clinical impact of the observed sex difference in comorbidity burden warrants further evaluation, our data highlight women with HIV as uniquely at-risk of multimorbidity and underscore the need to further characterize which modifiable factors mediate incremental risk in comorbidity onset and progression. This could pave the way for aging-related multimorbidity screening and prevention tools and strategies to be developed and appropriately tailored, with the goal of mitigating comorbidity effects that may accumulate and compound across the lifespan.

In our study, HIV modified the association of sex on aging-related NACM burden, a result that was attenuated (although remained significant) when adjusting for traditional comorbidity risk factors (eg, BMI, substance use, SES). These findings underscore the importance of prioritizing available, evidence-based interventions on traditional risk factors, such as tobacco cessation, to minimize NACM risk and progression among PWH; and furthermore, suggest that the optimal timing and impact of risk-modification efforts may differ by sex and gender in this population.^29,30^ WIHS participants primarily included women of color living in poverty compared with MACS participants representing predominantly White men with elevated SES (Table 1). The higher NACM burden observed in women vs men, particularly among PWH, could be related in part to the imbalance in social determinants of health by sex and gender, including race and factors not fully accounted for in measures assessed such as caregiver responsibilities, access to care, and structural racism.^31,32^ Interestingly, while we observed a higher NACM burden among non-Hispanic White vs Black participants in unadjusted and adjusted analyses, women had a higher comorbidity burden than men across racial and ethnic groups (eFigure 2 in Supplement 1). These data highlight the complex interplay of sex and gender, race, and social determinants of health in mediating health outcomes as evidenced by mixed data among PWH regarding the association of race with comorbidity burden^11,33^—differences in study findings may be related to differential representation of sex and gender, racial and ethnic minority groups, geographic regionality, and health care access, as well as which individual comorbidities were ascertained and included, among other factors. Finally, sex-differential factors not discretely adjusted for in our analysis could have contributed to observed differences in NACM burden, including stigma and stress associated with belonging to a sexual minority group (men who have sex with men).^34^

How psychosocial-biologic pathways may converge to increase multimorbidity risk in PWH, especially for women and people in impoverished communities, is an area of active investigation. Social determinants of health and trauma history have been linked with chronic inflammation via activation of stress responses, which in turn affects comorbidity risk, potentially exacerbated in PWH and women.^35,36,37^ Among PWH, ongoing systemic inflammation and immune dysregulation despite ART is associated with NACM development.^38,39^ Women with HIV have higher levels of inflammation and immune activation than men with HIV, even after achieving HIV-1 suppression, which may contribute to sex differences in NACM.^40,41^ Other possible mechanisms mediating sex differences in multimorbidity prevalence and composition in PWH include differences in gut permeability leading to microbial translocation^42^; sex hormone effects including during the menopausal transition^43^; and microvasculopathy, a condition with multisystem end-organ impact that disproportionately affects women.^44^ Furthermore, ongoing viral replication despite HIV treatment, either in reservoir sites or in the form of low level viremia (described as “blips”) due to incomplete ART adherence, may link HIV-associated inflammation and comorbidity risk.^39,45^ Among women with HIV in the WIHS, cumulative HIV-1 viremia after ART initiation increased the risk of multimorbidity in a dose-dependent manner^46^; data suggest that interventions addressing mental health, sociobehaviorial, and structural factors are urgently needed to support women at greatest risk of HIV-1 viremia.^47^

The individual comorbidities driving overall NACM burden significantly differed by sex, as did the criteria by which women vs men met a particular NACM definition, emphasizing the importance of considering sex-specific factors in comorbidity pathogenesis and risk. For example, 38% vs 10% of women vs men had prevalent lung disease with the vast majority of women meeting criteria by reported asthma diagnosis; this likely reflects the higher proportion of Black race, cigarette use, and lower SES (all established asthma risk factors) represented among WIHS vs MACS participants.^48^ Consistent with previous reports in PWH, we found higher prevalent bone disease and diabetes in women than men, highlighting sex-specific pathways conferring differential NACM risk that could be targeted for intervention (eg, addressing elevated BMI to mitigate risk of metabolic-related NACM in women).^9,49^ The higher prevalence of hypertension, dyslipidemia, liver disease, psychiatric illness, and non-AIDS cancer among men vs women is likely multifactorial, related to the older age of MACS participants, increased likelihood of care access or engagement (majority met criteria by reported medication use), and/or historic ART use with potentially cardiotoxic agents, eg, protease inhibitors.

Existing comorbidity risk-assessment tools developed in the general population focus on individual conditions, such as cardiovascular disease and fracture, and underestimate risk in PWH.^50,51,52,53^ Inaccurate performance of such algorithms in PWH, and particularly among women, may be associated with social determinants of health not being comprehensively captured nor HIV indices included, such as cumulative HIV-1 nonsuppression, which has been shown in this analysis and others to increase the risk of specific NACM, multimorbidity, and death.^46,54,55^ Our study supports development of innovative, holistic approaches to promote healthy aging in HIV that prioritize screening and prevention of multimorbidity, integrate assessment of traditional and HIV-related factors, and consider sociobiologic influences of sex and gender. Future studies should also evaluate the optimal timing to commence aging-related comorbidity risk-assessment and intervention, which likely differs by sex and gender, HIV serostatus, and other social determinants of health.^14,26,56^

### Strengths and Limitations

Strengths of this analysis included use of well-curated data from participants with semiannual study visits over 2 to 3 decades; equal representation of women and men allowing for adequately powered sex-stratified analyses; assessment of ten robustly defined NACM allowing for comprehensive assessment of multimorbidity; and inclusion of SES in adjusted models. Furthermore, as current HIV Primary Care Guidance does not specifically address managing multimorbidity, our findings provide insights into research priority areas to address these gaps.^57^

Limitations to this study included differences in participant characteristics and NACM risk by sex that could have been related to differential WIHS/MACS recruitment and enrollment criteria or study observation length. While we were not able to control for unmeasured confounders, even after adjusting for age and other traditional comorbidity risk factors including SES, our primary finding that women had a higher NACM burden than men remained consistent. Our participant sample may not be demographically representative of all PWH in the US; thus, findings by sex or gender should be interpreted in this context. While cross-sectional methods maximized the opportunity for WIHS/MACS participants to meet criteria for each NACM, we were not able to account for the contribution of time-varying covariates such as longitudinal viremia, the menopausal transition, patterns of and/or cumulative ART use, and use of NACM-specific therapies. We robustly defined NACM using published criteria^15,16^ and up to 3 data sources; however, some definitions relied solely on self-reported medication use or diagnosis which is subject to bias, especially if participants accessed the health care system differently. Furthermore, the degree of difference in NACM burden by sex (3.4 vs 3.2 among women vs men) was statistically significant. However, the clinical impact on quality of life, health care utilization and cost, and mortality has yet to be determined and should be prioritized in future study.

## Conclusions

Our data presented herein and previously published highlight PWH as an aging population at unique risk of multimorbidity with women disproportionately affected.^15,16^ Our findings underscore the need to accurately identify PWH at risk of multimorbidity to offer timely and tailored risk-modification interventions, with consideration given to sex and gender, so that outcomes can be optimized in this population.

## Notes

Supplement 1. **eTable 1.** Definitions of non-AIDS Comorbidities (NACM) and Criteria Met by the Overall Cohort and Stratified by Sex **eTable 2.** Categorization of Socioeconomic Status Based on Education, Income, and Federal Poverty Level Among WIHS and MACS Cohort Study Participants **eTable 3.** The Prevalence of non-AIDS Comorbidities (NACM) Among Women and Men at Last Observation in the WIHS and MACS Cohort Studies **eTable 4.** Age-Stratified Prevalence and Burden of non-AIDS Comorbidities (NACM) Among Women and Men at Last Observation in the WIHS and MACS Cohort Studies **eTable 5.** Estimated Mean Difference in non-AIDS Comorbidity (NACM) Burden Among Persons Living With HIV at Last Observation in the WIHS and MACS Cohort Studies **eTable 6.** Univariable Analysis of Risk Factors at Last Observation Associated With Prevalent Burden of non-AIDS Comorbidities (NACM) Among Persons Living With HIV in the WIHS and MACS Cohort Studies **eFigure 1.** Study Flow Diagram of Women’s Interagency HIV Study (WIHS) and Multicenter AIDS Cohort Study (MACS) Participants Included in the Analysis **eFigure 2.** Distribution of Prevalent non-AIDS Comorbidity (NACM) Burden by Racial and Ethnic Group, Sex, and Age Group Overall and Stratified by HIV Serostatus

Supplement 2.
Data Sharing Statement
