# Supplementary material for: Aging-Related Comorbidity Burden Among Women and Men With or At-Risk for HIV in the US, 2008-2019
Source: JAMA Netw Open. 2023 Aug 7;6(8):e2327584. doi: 10.1001/jamanetworkopen.2023.27584 (PMC10407688; doi:10.1001/jamanetworkopen.2023.27584)
Supplement: Supplement 1. — eTable 1. Definitions of non-AIDS Comorbidities (NACM) and Criteria Met by the Overall Cohort and Stratified by Sex eTable 2. Categorization of Socioeconomic Status Based on Education, Income, and Federal Poverty Level Among WIHS and MACS Cohort Study Participants eTable 3. The Prevalence of non-AIDS Comorbidities (NACM) Among Women and Men at Last Observation in the WIHS and MACS Cohort Studies eTable 4. Age-Stratified Prevalence and Burden of non-AIDS Comorbidities (NACM) Among Women and Men at Last Observation in the WIHS and MACS Cohort Studies eTable 5. Estimated Mean Difference in non-AIDS Comorbidity (NACM) Burden Among Persons Living With HIV at Last Observation in the WIHS and MACS Cohort Studies eTable 6. Univariable Analysis of Risk Factors at Last Observation Associated With Prevalent Burden of non-AIDS Comorbidities (NACM) Among Persons Living With HIV in the WIHS and MACS Cohort Studies eFigure 1. Study Flow Diagram of Women’s Interagency HIV Study (WIHS) and Multicenter AIDS Cohort Study (MACS) Participants Included in the Analysis eFigure 2. Distribution of Prevalent non-AIDS Comorbidity (NACM) Burden by Racial and Ethnic Group, Sex, and Age Group Overall and Stratified by HIV Serostatus [file jamanetwopen-e2327584-s001.pdf]

Supplemental Online Content

Collins LF, Palella FJ Jr, Mehta CC, et al. Aging-related comorbidity burden among women and men with or at-risk for HIV in the US, 2008-2019. *JAMA Netw Open.* 2023;6(8):e2327584. doi:10.1001/jamanetworkopen.2023.27584

eTable 1. Definitions of non-AIDS Comorbidities (NACM) and Criteria Met by the Overall Cohort and Stratified by Sex

eTable 2. Categorization of Socioeconomic Status Based on Education, Income, and Federal Poverty Level Among WIHS and MACS Cohort Study Participants

eTable 3. The Prevalence of non-AIDS Comorbidities (NACM) Among Women and Men at Last Observation in the WIHS and MACS Cohort Studies

eTable 4. Age-Stratified Prevalence and Burden of non-AIDS Comorbidities (NACM) Among Women and Men at Last Observation in the WIHS and MACS Cohort Studies

eTable 5. Estimated Mean Difference in non-AIDS Comorbidity (NACM) Burden Among Persons Living With HIV at Last Observation in the WIHS and MACS Cohort Studies

eTable 6. Univariable Analysis of Risk Factors at Last Observation Associated With Prevalent Burden of non-AIDS Comorbidities (NACM) Among Persons Living With HIV in the WIHS and MACS Cohort Studies

eFigure 1. Study Flow Diagram of Women's Interagency HIV Study (WIHS) and Multicenter AIDS Cohort Study (MACS) Participants Included in the Analysis

eFigure 2. Distribution of Prevalent non-AIDS Comorbidity (NACM) Burden by Racial and Ethnic Group, Sex, and Age Group Overall and Stratified by HIV Serostatus

This supplemental material has been provided by the authors to give readers additional information about their work.

© 2023 Collins LF et al. *JAMA Network Open.*

eTable 1. Definitions of non-AIDS comorbidities (NACM) and criteria met by the overall cohort and stratified by sex

	NACM Criteria Met*		
NACM definition	**Total (n=5929)**	**Women (n=3238)**	**Men (n=2691)**
**Hypertension**	**4214 (71)**	**2188 (68)**	**2026 (75)**
Systolic BP ≥140 or diastolic BP ≥90 on any two study visits	3601 (61)	1761 (54)	1840 (68)
<u>*Or*</u> self-report of anti-hypertensive medication	3280 (55)	1827 (56)	1453 (54)
**Psychiatric illness**	**3336 (56)**	**1771 (55)**	**1565 (58)**
Self-report of depression, anxiety, or psychiatric problem	3820 (64)	2162 (67)	1658 (62)
<u>*And*</u> of anti-depressant/-psychotic medication	4004 (68)	1900 (59)	2104 (78)
<u>*Or*</u> *CES-D* ≥16 on two consecutive study visits	1261 (21)	690 (21)	571 (21)
**Dyslipidemia**	**3040 (51)**	**1312 (41)**	**1728 (64)**
LDL ≥130 mg/dL and HDL <40 mg/dL	1294 (22)	488 (15)	806 (30)
<u>*Or*</u> self-report of lipid-lowering medication	2575 (43)	1091 (34)	1484 (55)
**Liver disease**	**2125 (36)**	**1093 (34)**	**1032 (38)**
Laboratory-confirmed chronic hepatitis B or C viral infection	840 (14)	455 (14)	385 (14)
<u>*Or*</u> FIB-4 score >3.25	922 (16)	486 (15)	436 (16)
<u>*Or*</u> APRI score >0.7	1822 (31)	970 (30)	852 (32)
**Bone disease**	**1876 (32)**	**1364 (42)**	**512 (19)**
Self-report of low bone density, osteopenia, or osteoporosis	602 (10)	502 (16)	100 (4)
<u>*Or*</u> of fracture	1173 (20)	773 (24)	400 (15)
<u>*Or*</u> of osteoporosis medication	867 (15)	758 (23)	109 (4)
**Lung disease**	**1504 (25)**	**1245 (38)**	**259 (10)**
Self-report of asthma	1432 (24)	1216 (38)	216 (8)
<u>*Or*</u> of chronic obstructive pulmonary disease or emphysema	175 (3)	122 (4)	53 (2)
**Diabetes, type 2**	**1233 (21)**	**763 (24)**	**470 (17)**
Self-report of anti-diabetic medication	483 (13)	490 (15)	253 (11)
<u>*Or*</u> FBG ≥126 mg/dL on any two study visits	811 (14)	391 (12)	420 (15)

© 2023 Collins LF et al. *JAMA Network Open.*

Or HgbA1c ≥6.5% and FBG ≥126 at one study visit			
**Cardiovascular disease**	**900 (15)**	**493 (15)**	**407 (15)**
Self-report of myocardial infarction or heart attack	288 (5)	155 (5)	133 (5)
Or of stroke, cerebrovascular accident, or transient ischemic attack	453 (8)	260 (8)	193 (7)
Or of surgery to open blocked vessels, revascularization, or angioplasty	384 (6)	211 (7)	173 (6)
Or of heart failure or cardiomyopathy	78 (1)	9 (<1)	69 (3)
**Chronic kidney disease**	**848 (14)**	**444 (14)**	**404 (15)**
eGFR[¶] <60 mL/min/1.73 m^2 on two consecutive study visits			
**Non-AIDS cancer**	**540 (9)**	**219 (7)**	**321 (12)**
Self-report of any of the following[†]:			
Lung	43 (1)	23 (1)	20 (1)
Colon	47 (1)	8 (<1)	39 (1)
Liver	24 (<1)	13 (<1)	11 (<1)
Hodgkin's lymphoma	12 (<1)	6 (<1)	6 (<1)
Breast	- - -	69 (2)	- - -
Uterine	- - -	24 (1)	- - -
Ovarian	- - -	11 (<1)	- - -
Prostate	- - -	- - -	96 (4)
Penile	- - -	- - -	8 (<1)
Testicular	- - -	- - -	4 (<1)
Other[‡]	287 (5)	104 (3)	183 (7)

Abbreviations: APRI = AST to Platelet Ratio Index; CES-D = Center for Epidemiologic Studies-Depression Scale; CKD-EPI = Chronic Kidney Disease Epidemiology Collaboration; eGFR = estimated Glomerular Filtration Rate; FBG = Fasting Blood Glucose; FIB-4 = Fibrosis-4; HDL = High-Density Lipoprotein; HgbA1c = Hemoglobin A1c; LDL = Low-Density Lipoprotein

*Data presented in the first entry of each row represent the total prevalence of the NACM listed for the cohort and then stratified by sex; the following entries per row are the number of respective individuals who met the specified NACM criteria and are not mutually exclusively

[¶]Determined by the CKD-epi formula

[†]Specifically excluded non-melanoma skin cancer, Kaposi's sarcoma, Non-Hodgkin's lymphoma, central nervous system lymphoma

[‡]Included cancer of the head and neck, eye, brain, thyroid, esophagus, stomach, bowel, anus or rectum, pancreas, kidney, bladder, bone as well as sarcoma and leukemia

© 2023 Collins LF et al. *JAMA Network Open.*

eTable 2. Categorization of socioeconomic status based on education, income, and federal poverty level among WIHS and MACS cohort study participants

Socioeconomic status, n (%)	**Total (n=5928*)**	**Women (n=3237)**	**Men (n=2691)**
(1) <HS, <150% FPL, unemployed	1594 (27)	1340 (41)	254 (9)
(2) <HS, either <150% FPL or unemployed	723 (12)	553 (17)	170 (6)
(3) Either <HS, <150% FPL or unemployed	1257 (21)	675 (21)	582 (22)
(4) >HS, either ≥150% FPL or employed	1027 (17)	340 (11)	687 (26)
(5) >HS, ≥150% FPL and employed	1327 (22)	329 (10)	998 (37)

Abbreviations: FPL = federal poverty level; HS = high school education

*n=1 missing from the cohort total as participant had incomplete data on elements included in the socioeconomic scale

© 2023 Collins LF et al. *JAMA Network Open.*

eTable 3. The prevalence of non-AIDS comorbidities (NACM) among women and men at last observation in the WIHS and MACS cohort studies

NACM, n (%)	**Total (n=5929)**	**Women (n=3238)**	**Men (n=2691)**	*P value**
Hypertension	4214 (71)	2188 (68)	2026 (75)	<0·001
Psychiatric illness	3336 (56)	1771 (55)	1565 (58)	0·008
Dyslipidemia	3040 (51)	1312 (41)	1728 (64)	<0·001
Liver disease	2125 (36)	1093 (34)	1032 (38)	<0·001
Bone disease	1876 (32)	1364 (42)	512 (19)	<0·001
Lung disease	1504 (25)	1245 (38)	259 (10)	<0·001
Diabetes, type 2	1233 (21)	763 (24)	470 (17)	<0·001
Cardiovascular disease	900 (15)	493 (15)	407 (15)	0·91
Chronic kidney disease	848 (14)	444 (14)	404 (15)	0·15
Cancer, non-AIDS	540 (9)	219 (7)	321 (12)	<0·001
**Mean NACM burden (sd)**	**3·31 (1·96)**	**3·36 (2·08)**	**3·24 (1·79)**	**0·02**

Abbreviation: sd = standard deviation

*Chi-square test performed for difference in NACM prevalence by sex and two-sample *t*-test performed for difference in mean NACM burden by sex

© 2023 Collins LF et al. *JAMA Network Open.*

eTable 4. Age-stratified prevalence and burden of non-AIDS comorbidities (NACM) among women and men at last observation in the WIHS and MACS cohort studies

	**Women (n=3238)**					**Men (n=2691)**				
NACM, n (%)	<40y (n=477)	40-49y (n=946)	50-59y (n=1265)	60-69y (n=485)	≥70y (n=65)	<40y (n=308)	40-49y (n=334)	50-59y (n=808)	60-69y (n=834)	≥70y (n=407)
HTN	172 (36·1)	536 (56·7)	969 (76·6)	446 (92·0)	65 (100·0)	95 (30·8)	189 (56·6)	626 (77·5)	733 (87·9)	383 (94·1)
Psych	182 (38·2)	467 (49·4)	767 (60·6)	319 (65·8)	36 (55·4)	110 (35·7)	193 (57·8)	516 (63·9)	525 (63·0)	221 (54·3)
DL	73 (15·3)	316 (33·4)	582 (46·0)	295 (60·8)	46 (70·8)	64 (20·8)	149 (44·6)	540 (66·8)	638 (76·5)	337 (82·8)
Liver	74 (15·5)	251 (26·5)	476 (37·6)	259 (53·4)	33 (50·8)	47 (15·3)	118 (35·3)	348 (43·1)	341 (40·9)	178 (43·7)
Bone	84 (17·6)	296 (31·3)	619 (48·9)	318 (65·6)	47 (72·3)	29 (9·4)	37 (11·1)	125 (15·5)	200 (24·0)	121 (29·7)
Lung	136 (28·5)	299 (31·6)	566 (44·7)	218 (45·0)	26 (40·0)	13 (4·2)	19 (5·7)	74 (9·2)	102 (12·2)	51 (12·5)
DM2	37 (7·8)	159 (16·8)	363 (28·7)	180 (37·1)	24 (36·9)	12 (3·9)	42 (12·6)	145 (18·0)	168 (20·1)	103 (25·3)
CVD	15 (3·1)	92 (9·7)	239 (18·9)	126 (26·0)	21 (32·3)	6 (2·0)	29 (8·7)	99 (12·3)	162 (19·4)	111 (27·3)
CKD	4 (0·8)	42 (4·4)	196 (15·5)	164 (33·8)	38 (58·5)	4 (1·3)	13 (3·9)	95 (11·8)	164 (19·7)	128 (31·5)
Cancer	5 (1·1)	39 (4·1)	103 (8·1)	59 (12·2)	13 (20·0)	4 (1·3)	10 (3·0)	75 (9·3)	131 (15·7)	101 (24·8)
					Total NACM count					
**Mean (sd)**	1·64 (1·38)	2·64 (1·79)	3·86 (1·90)	4·92 (1·84)	5·37 (1·83)	1·25 (1·18)	2·39 (1·48)	3·27 (1·55)	3·79 (1·65)	4·26 (1·66)
**Median (Q1-Q3)**	1 (1-2)	2 (1-4)	4 (2-5)	5 (4-6)	5 (4-7)	1 (0-2)	2 (1-3)	3 (2-4)	4 (3-5)	4 (3-5)

Abbreviations: CKD = chronic kidney disease; CVD = cardiovascular disease; DL = dyslipidemia; DM2 = diabetes, type 2; HTN = hypertension; Psych = psychiatric illness; sd = standard deviation

© 2023 Collins LF et al. *JAMA Network Open.*

eTable 5. Estimated mean difference in non-AIDS comorbidity (NACM) burden among persons living with HIV at last observation in the WIHS and MACS cohort studies

Estimated Mean Difference (95% CI) in NACM Burden

**Women versus Men with HIV**				
	**MODEL 3***		**MODEL 4†**	
Age (yrs)		*P value*		*P value*
<40	0·33 (0·03, 0·63)	0·03	0·03 (-0·30, 0·36)	0·86
40-49	0·37 (0·11, 0·62)	0·005	0·11 (-0·16, 0·39)	0·41
50-59	0·38 (0·19, 0·56)	<0·001	0·28 (0·08, 0·48)	0·007
60-69	0·66 (0·42, 0·90)	<0·001	0·60 (0·32, 0·87)	<0·001
≥70	0·62 (0·06, 1·18)	0·03	0·52 (-0·08, 1·11)	0·09

*Model 3: Linear regression model including age (*p*<0·001), sex (*p*<0·001), and the age*sex interaction term (*p*=0·29) in the model.

†Model 4: Adjusted linear regression model including the following covariates in the model: age (*p*<0·001), sex (*p*<0·001), the age*sex interaction term (*p*=0·04), race (*p*<0·001), body mass index (*p*<0·001), socioeconomic status (*p*<0·001), cigarette use (*p*<0·001), current alcohol use (*p*=0·05), crack/cocaine use (*p*<0·001), current CD4 count (*p*=0·59), CD4 nadir (*p*=0·65), proportion of visits HIV suppressed from initial MACS/WIHS visit (*p*<0·001), time since antiretroviral therapy initiation (*p*<0·001), abacavir use in last 6 months (*p*<0·001), and protease inhibitor use in last 6 months (*p*=0·07). The model uses n=3236 observations with complete data.

© 2023 Collins LF et al. *JAMA Network Open.*

eTable 6. Univariable analysis of risk factors at last observation associated with prevalent burden of non-AIDS comorbidities (NACM) among persons living with HIV in the WIHS and MACS cohort studies

Risk factor	Estimated mean number of NACM (95% CI)	Beta (±SE)*	*P* value[†]
Sex			0·08
Women	3·51 (3·43, 3·60)	0·12 (±0·07)	
Men	3·40 (3·29, 3·50)	Ref	
Age group, *yrs*			<0·001
≥70	5·27 (5·01, 5·53)	3·74 (±0·15)	
60-69	4·65 (4·53, 4·77)	3·13 (±0·10)	
50-59	3·77 (3·68, 3·85)	2·24 (±0·09)	
40-49	2·73 (2·61, 2·84)	1·20 (±0·10)	
<40	1·52 (1·37, 1·67)	Ref	
Race			<0·001
Black, non-Hispanic	3·37 (3·28, 3·46)	-0·56 (±0·08)	
Hispanic/other	3·12 (2·98, 3·25)	-0·81 (±0·09)	
White, non-Hispanic	3·93 (3·81, 4·05)	Ref	
Body mass index, *kg/m²*			0·20
≥30	3·50 (3·42, 3·59)	0·09 (±0·07)	
<30	3·42 (3·31, 3·52)	Ref	
Socioeconomic status[¶]			<0·001
Level 1 (low)	3·93 (3·82, 4·04)	0·97 (±0·09)	
Level 2	2·71 (2·54, 2·88)	-0·25 (±0·11)	
Level 3	3·65 (3·52, 3·78)	0·69 (±0·10)	
Level 4	3·55 (3·38, 3·71)	0·58 (±0·11)	
Level 5 (high)	2·97 (2·82, 3·11)	Ref	
Cigarette use			<0·001
Current	3·68 (3·57, 3·80)	0·75 (±0·08)	
Former	3·77 (3·67, 3·88)	0·84 (±0·08)	
Never	2·93 (2·82, 3·04)	Ref	
Current alcohol use			<0·001
>7 drinks/week	3·40 (3·19, 3·61)	-0·37 (±0·07)	
1-7 drinks/week	3·27 (3·18, 3·37)	-0·24 (±0·12)	
None	3·64 (3·55, 3·74)	Ref	
Crack/cocaine use			<0·001
Current	3·48 (3·24, 3·72)	0·39 (±0·13)	
Former	3·96 (3·86, 4·06)	0·88 (±0·07)	
Never	3·09 (3·00, 3·17)	Ref	
CD4 count, *cells/mm³*			<0·001
<500	3·68 (3·56, 3·79)	0·30 (±0·07)	
≥500	3·38 (3·30, 3·46)	Ref	

© 2023 Collins LF et al. *JAMA Network Open.*

CD4 nadir, *cells/mm^3*			<0·001
<200	3·75 (3·64, 3·87)	0·39 (±0·07)	
≥200	3·36 (3·29, 3·87)	Ref	
Time since ART initiation			<0·001
≥15	4·17 (4·08, 2·95)	1·74 (±0·09)	
10 to <15	3·44 (3·30, 3·57)	1·01 (±0·11)	
5 to <10	2·83 (2·70, 2·95)	0·40 (±0·10)	
<5 or never initiated ART	2·42 (2·26, 2·58)	Ref	
Proportion visits HIV suppressed[‡] from initial study visit			<0·001
<90%	3·71 (3·64, 3·78)	0·87 (±0·07)	
≥90%	2·84 (2·72, 2·96)	Ref	
PI use in last 6 months			<0·001
Yes	3·72 (3·60, 3·84)	0·36 (±0·07)	
No	3·37 (3·29, 3·44)	Ref	
Abacavir use in last 6 months			<0·001
Yes	3·95 (3·81, 4·10)	0·60 (±0·08)	
No	3·36 (3·29, 3·43)	Ref	

Abbreviations: ART = antiretroviral therapy; HS = high school education; MACS = Multicenter AIDS Cohort Study; PI = protease inhibitor; SE = standard error; WIHS = Women's Interagency HIV Study

*Estimated mean difference in non-AIDS comorbidity burden

[†]Separate unadjusted linear regression for each covariate listed

[¶]Socioeconomic status determined by participant's reported educational level, federal poverty level, and employment status; graded from Level 1 (low) to Level 5 (high)

[‡]HIV viral load <200 copies/ml and/or <lower limit of quantification of assay

© 2023 Collins LF et al. *JAMA Network Open.*

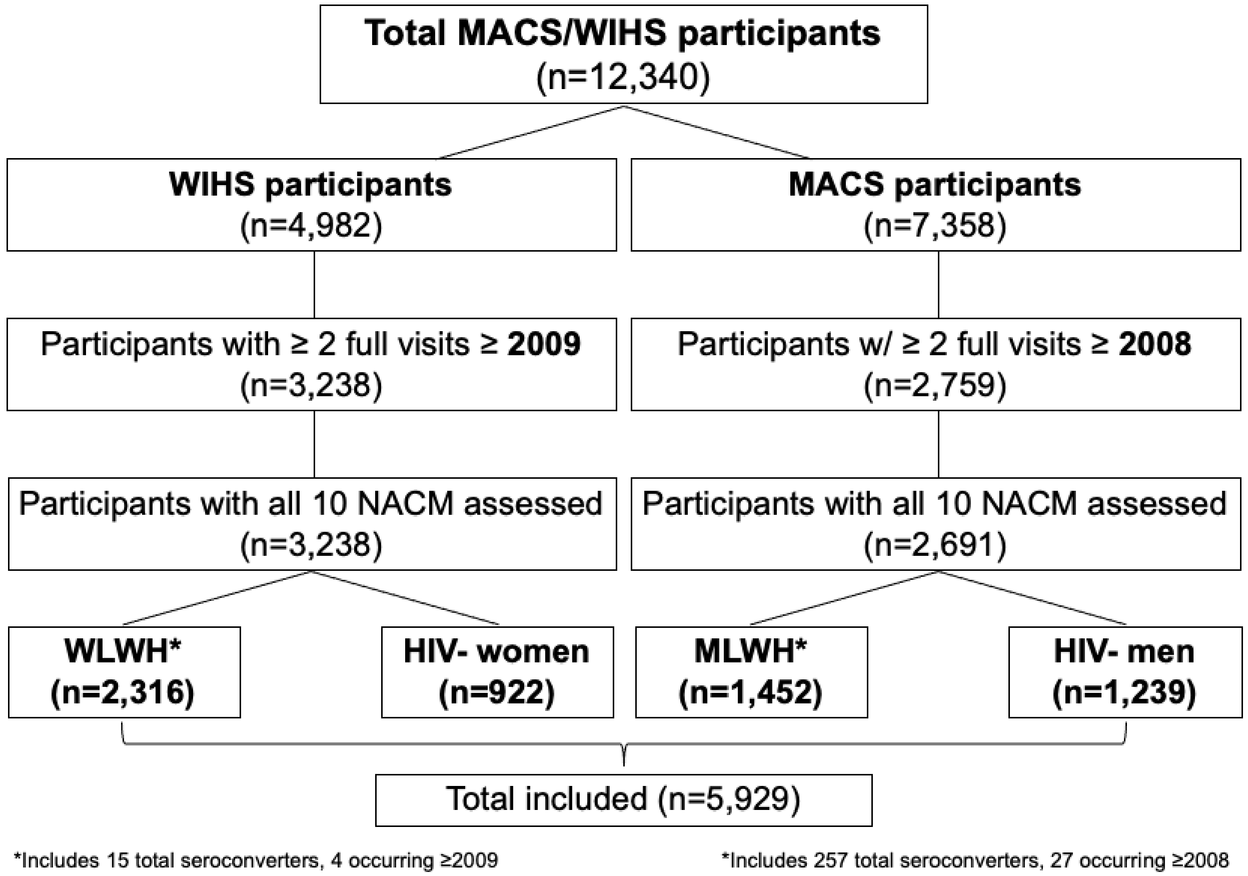

eFigure 1. Study flow diagram of Women's Interagency HIV Study (WIHS) and Multicenter AIDS Cohort Study (MACS) participants meeting inclusion criteria requiring follow-up after the calendar time in which >80% of participants with HIV reported antiretroviral therapy use (2009 and 2008, respectively) with observation through 03/2019. *Abbreviations: NACM = non-AIDS comorbidities; MLWH = men living with HIV; WLWH = women living with HIV.*

© 2023 Collins LF et al. *JAMA Network Open.*

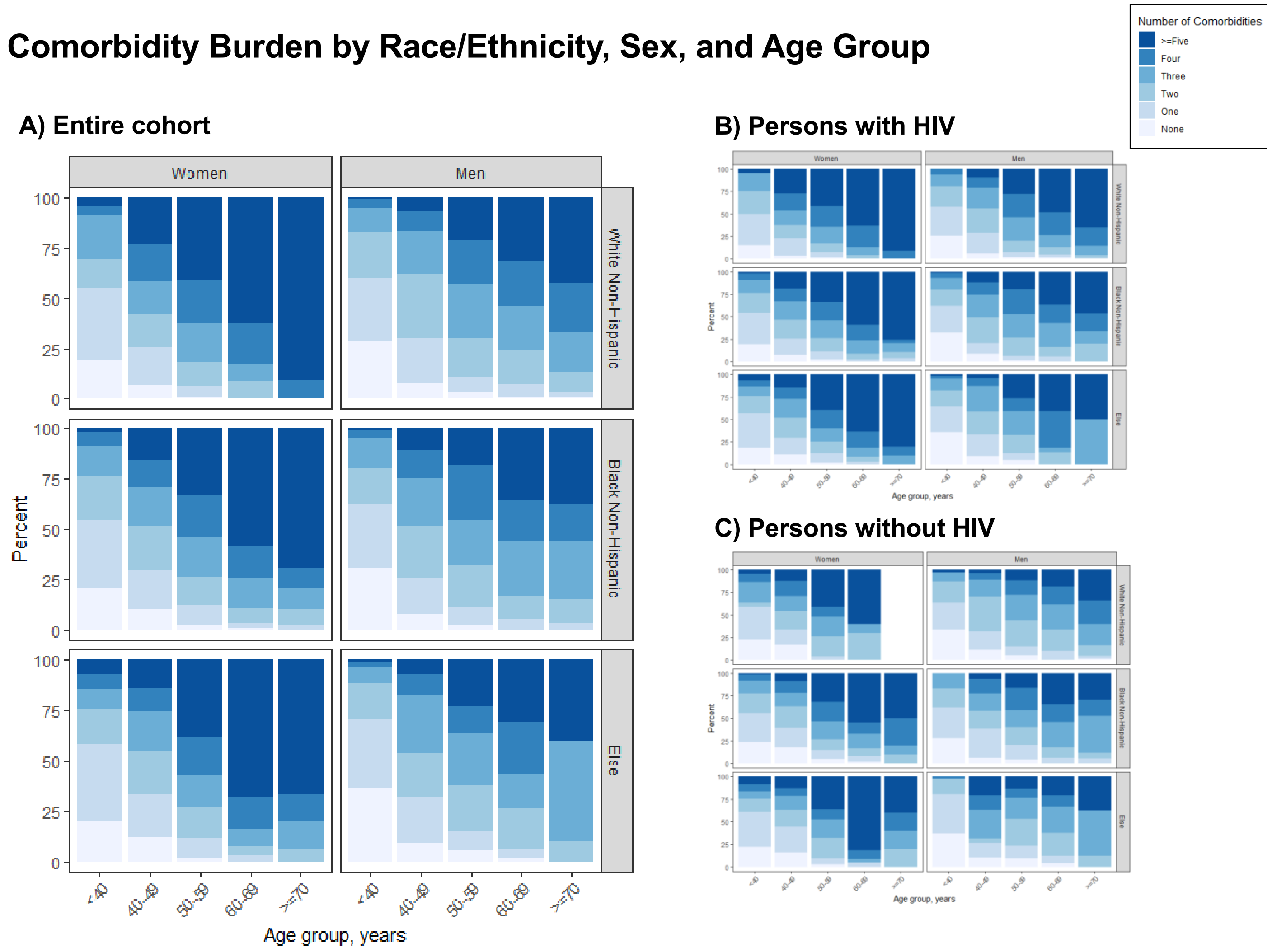

eFigure2. Distribution of prevalent non-AIDS comorbidity burden by racial/ethnic group, sex, and age group overall and stratified by HIV serostatus. Higher comorbidity burden for women versus men was observed in all racial/ethnic groups in the cohort overall (panel A) and specifically among persons with HIV (panel B) and persons without HIV (panel C).

Note: There are no Non-Hispanic women without HIV aged ≥70 years old.

© 2023 Collins LF et al. *JAMA Network Open.*
