# Supplementary material for: Aging-Related Comorbidity Burden Among Women and Men With or At-Risk for HIV in the US, 2008-2019
Source: JAMA Netw Open. 2023 Aug 7;6(8):e2327584. doi: 10.1001/jamanetworkopen.2023.27584 (PMC10407688; doi:10.1001/jamanetworkopen.2023.27584)
Supplement: Supplement 2. — Data Sharing Statement [file jamanetwopen-e2327584-s002.pdf]

Data Sharing Statement

Collins. Aging-Related Comorbidity Burden Among Women and Men With or At-Risk for HIV in the US, 2008-2019. *JAMA Netw Open*. Published August 07, 2023.
doi:10.1001/jamanetworkopen.2023.27584

Data
Data available: Yes
Data types: Other (please specify)
Additional Information: Please reference data sharing statement included in manuscript
How to access data: Please reference data sharing statement included in manuscript
When available: With publication

Supporting Documents
Document types: None

Additional Information
Who can access the data: Researchers requesting the data for analysis by contacting the MACS/WIHS combined cohort study data analysis and coordinating center (DACC) for approval
Types of analyses: See above
Mechanisms of data availability: See above
